# Indian Phenotype Characteristics Among Patients with Type 2 Diabetes Mellitus: Insights from a Non-interventional Nationwide Registry in India

**DOI:** 10.17925/EE.2022.18.1.63

**Published:** 2022-05-30

**Authors:** Sanjay Kalra, Ambrish Mithal, Abdul Hamid Zargar, Bipin Sethi, Mala Dharmalingam, Sujoy Ghosh, Ranjini Sen

**Affiliations:** 1. Department of Endocrinology, Bharti Hospital, Karnal, India; 2. Department of Endocrinology, Max Healthcare, Saket, India; 3. Centre for Diabetes and Endocrine Care, Gulshan Nagar, Srinagar, India; 4. Department of Endocrinology, CARE Super Specialty Hospital & Transplant Centre, Hyderabad, India; 5. Department of Endocrinology, Ramaiah Medical College, Bengaluru, India; 6. Department of Endocrinology, Institute of Post-Graduate Medical Education and Research and Seth Sukhlal Karnani Memorial Hospital, Kolkata, India; 7. AstraZeneca Pharma India Ltd, Bengaluru, India

**Keywords:** Body fat percentage, body mass index, glycated haemoglobin A, obesity, overweight, type 2 diabetes mellitus

## Abstract

**Background**: Indian patients with type 2 diabetes mellitus (T2D) constitute one-sixth of affected adults globally. Here, we evaluate the association of body mass index (BMI) with body fat percentage (BF%) and glycated haemoglobin (HbA1c) levels among patients with T2D in India. **Method**: This was a cross-sectional Indian registry study across 845 geographically diverse zones between December 2017 and August 2019. **Results**: Of 37,927 patients, 55.6% were men, with a mean ± standard deviation age of 54.2 ± 11.5 years and HbA1c of 8.3 ± 1.71%. Mean ± standard deviation BMI and BF% were 27.0 ± 4.6 kg/m2 and 32.0 ± 8.0%, respectively. Overall, 15.4% of patients were overweight, and 25.0% were obese. Despite fewer males (20.7%) having BMI-based obesity than females (31.2%), around three-quarters of both sexes had BF%-defined obesity (males 77.2%; females 71.2%). One-third of males (34.6%) and 41.9% of females had BF%-defined obesity despite normal BMI. The association was substantiated by a moderately significant correlation (r=0.51) between BMI and BF% in the overall population (p<0.0001). **Conclusion**: This pan-India registry presents a real-world reflection of the Asian Indian phenotype: high BF% despite lower BMI in people with T2D. This highlights the importance of primordial and primary prevention, and may guide decisions on the choice of agents for glycaemic control, with a preference for drugs that promote weight loss or are weight neutral.

The pandemic of type 2 diabetes mellitus (T2D) is a growing concern, especially in low- and middle-income countries, which contribute to nearly 75% of the disease burden.^1^ Indian patients with T2D constitute 1 in 6 adults with T2D globally, with marked differences in prevalence across the states.^2,3^ The younger age of onset and faster progression from prediabetes to diabetes among Indians increases the disease burden.^4^ With a 10.4% age-adjusted comparative prevalence of T2D, India accounts for the highest mortality in the Southeast Asian region, with 1,010,262 deaths due to T2D in 2019.^2,5^ The age-standardized disability-adjusted life year rate for T2D increased in India by 39.6% (95% uncertainty interval [UI] 32.1– 46.7%) from 1990 to 2016.^5^ Notably, of patients who died due to T2D in India in 2016, 42.6% (95% UI 41.6– 43.9%) were younger than 70 years.^5^ Nearly half (47.3%) of the patients diagnosed with diabetes had not been diagnosed previously.^6^

Although the prevalence of T2D remains higher in the economically advanced states in India, it has surged rapidly in the less-developed states.^5^ Rapid epidemiological transition with an ageing population, compounded by modifiable risk factors such as an unhealthy diet, sedentary lifestyle, tobacco use and obesity, is an important driver of the T2D epidemic in India.^5^ Among these, obesity is one of the most pivotal and dominant risk factors; prevalence of overweight in India markedly increased from 9.0% in 1990 to 20.4% in 2016.^5^ Anthropometric analysis from the National Family Health Survey III and IV highlighted a rising prevalence of overweight/obesity across urban and rural locations – the prevalence among men and women was observed to be 38.4% and 36.2%, respectively.^7^ A systematic review reported that more than 135 million individuals are affected by obesity in India, with variations in prevalence rates of obesity and central obesity (11.8–31.3% and 16.9–36.3%, respectively).^8^ It is estimated that the percentage of overweight people will more than double and obesity will triple among Indian adults between 2010 and 2040.^9^ Excessive accumulation of visceral fat causes an imbalance in endocrine function and release of proinflammatory factors, which results in the development of insulin resistance, T2D and other poor cardiometabolic outcomes.^10,11^

The Asian Indian phenotype (IP) is characterized by unique clinical and biochemical abnormalities, including increased insulin resistance and greater abdominal adiposity (i.e. higher waist circumference and waist-to-hip ratio), despite lower body mass index (BMI), lower adiponectin and higher high-sensitivity C-reactive protein levels. These, together with the dyslipidaemia triad – low high-density lipoprotein (HDL), high low-density lipoprotein (LDL) and high triglycerides – make Indians more prone to developing T2D.^12,13^ South Asians tend to have a higher body fat percentage (BF%) compared with other ethnicities, despite lower BMI values – commonly referred to as the Yajnik and Yudnik (Y-Y) paradox.^14,15^ Evaluating the body composition in terms of BF% in patients with T2D can identify risk factors, facilitating early prevention and reducing complications. Additionally, because of the heterogeneity of T2D among the states in India, it is vital to understand the link between risk factors and BF%. Previous studies from India have explored the relationship between BMI and BF%; however, there are gaps in evidence, as the studies had a limited sample size.^16–23^ In this regard, large-scale registries can provide robust data on IP attributes in people with diabetes.

This multistate IP registry aimed to evaluate the BF% across various BMI categories in patients with T2D in India. As secondary objectives, the study aimed to analyse patient characteristics, correlate glycated haemoglobin (HbA1c) levels with various BMI categories, record associated comorbidities, and document use of on-going glucose-lowering drugs.

## Methods

### Study design and setting

We conducted this non-interventional, multicentre, cross-sectional study across 845 study centres from geographically diverse zones of India, encompassing different tiers of healthcare centres and investigators (general practitioners and specialists in diabetes management) between 11 December 2017 and 8 August 2019. Participants were randomly recruited across India, without any distinct zone variation, predominantly from the urban centres. The study was initiated at each site after ethics committee approval, and was conducted in accordance with the Declaration of Helsinki, International Council for Harmonisation Good Clinical Practice, and Good Pharmacoepidemiology Practice guidelines. All patients provided written informed consent for participation before enrolment during their routine clinic visits. Adults (≥18 years old) with previously diagnosed T2D with an HbA1c report available within the 3 months prior to screening were included in the registry. Patients with type 1 diabetes, pancreatic diabetes or secondary diabetes were excluded. No study medication was prescribed or administered as part of study procedures.

### Data collection and data variables

Information was collected on demographics (age, sex, history of tobacco use), anthropometry (weight, height, waist and hip circumference), clinical characteristics (vital signs, duration of T2D, comorbidities such as hypertension, dyslipidaemia, chronic kidney disease, cardiovascular disease [CVD], heart failure, stroke/transient ischaemic attack, neuropathy, retinopathy), HbA1c and anti-diabetic/concomitant medications. T2D was defined as HbA1c >6.5% and fasting blood glucose >120 mg/dL. Body fat analysis included total body fat content and distribution, measured using a validated Omron fat analyzer (model HBF-212; Omron Healthcare India Pvt. Ltd., Gurugram, Haryana, India). In addition to weight, it also measured the BF%, visceral fat level and BMI. Asian Indian cut-off values for defining obesity were used in this study; operational definitions of data variables are presented in *Table 1*.^15,19–21^ Normal weight obesity (NWO) was defined as having normal body weight but with a high BF%, and leads to some of the same health risks as obesity; metabolically obese patients with normal weight were defined as those with normal weight and BMI, but displaying some metabolic characteristics that increase the risk of developing metabolic syndrome in the same way as obesity.

**Table 1:** Operational definitions of classifications^15,19–21^

Variable	Classification
BMI, kg/m^2 a,b^,^15,19^	Underweight: <18.5 Normal: 18.5-22.9 Overweight: 23.0-24.9 Pre-obese: 25.0-29.9 Obese: ≥30.0 Type 1 (obese): 30.0-40.0 Type 2 (morbidly obese): 40.1≥50.0 Type 3 (super obese): >50.0
Body fat percentage, %^a,19^	
Male	Essential fat: 2–5 Athletes: 6–13 Fitness: 14–17 Acceptable: 18–24 Obese: ≥25
Female	Essential fat: 10–13 Athletes: 14–20 Fitness: 21–24 Acceptable: 25–31 Obese: ≥32
HbA1c, %^c,20,21^	<7.0 ≥7.0
Age groups, years	18–29 30–39 40–49 50 –59 ≥60
Duration of diabetes, years	<5 5–10 >10–20 >20

*^a^Since pre-obese criteria are not mentioned as part of the World Health Organization guidelines, online portals with information on Asian adults have been used.^19^*

*^b^Classification based on BMI.*

*^c^Analysis has been done in 4 categories.*

*BMI = body mass index; HbA1c = glycated haemoglobin.*

### Statistical analysis

The patient characteristics and variables were described using frequency distributions and proportions for categorical variables. Continuous variables were described using mean ± standard deviation (SD). To understand the effect of sex, subgroup analysis for males and females was conducted for anthropometric variables. Correlation between BF% and BMI was evaluated using the Pearson correlation coefficient (r). Statistical analyses were performed with statistical software, SAS^®^ 9.4 (SAS Institute Inc., Cary, NC, USA), and a p value of <0.05 was considered statistically significant.

**Table 2:** Sociodemographic characteristics of patients enrolled in the Indian Phenotype Registry

Variables	(N=37,927)
Age, years	
18–29	567 (1.5)
30–39	3,496 (9.2)
40–49	8,564 (22.6)
50–59	12,241 (32.3)
60 and above	13, 054 (34.4)
Sex	
Male	21,098 (55.6)
Female	16,827 (44.4)
History of tobacco use	
Yes	5,228 (13.8)
Medical/surgical history	
Yes	25,577 (67.4)
Details of medical history^a^	
Hypertension	18,225 (71.3)
Dyslipidaemia	12,887 (50.4)
CKD	698 (2.7)
CVD	2,347 (9.2)
Heart failure	472 (1.8)
Stroke/TIA	502 (2.0)
Neuropathy	2,393 (9.4)
Retinopathy	319 (1.2)
Other	5,599 (21.9)

*Values are n (%).*

*Missing data: age (n=2), sex (n=2), tobacco history (n=35), relevant medical/surgical history (n=58), details of medical history (n=45).*

*^a^Percentage calculated based on the number of subjects who had any relevant medical history.*

*CKD = chronic kidney disease; CVD = cardiovascular disease; TIA = transient ischaemic attack.*

## Results

Of 38,849 subjects with T2D enrolled in the registry, 37,927 were considered for the final analysis after excluding extreme or erroneous values.

### Sociodemographic characteristics

The mean age of patients was 54.2 ± 11.5 years; 54.9% (n=20,805) were aged 40–59 years and about one third (34.4%, n=13,054) were aged ≥60 years. More than half (55.6%; n=21,098) were men, and approximately 13.8% (n=5,228) were current tobacco users. In all, 67.4% (n=25,577) of patients had a medical/surgical history, of which hypertension (71.3%, n=18,225) and dyslipidaemia (50.4%, n=12,887) were most common. Furthermore, CVD and neuropathy were reported in 9.2% (n=2,347) and 9.4% (n=2,393) of patients, respectively (*Table 2*). The proportion of patients on concomitant medications was as follows: angiotensin II antagonists (47.6%, n=12,871), beta-blockers (14.8%, n=4,012), calcium channel blockers (21.2%, n=5,725), antithrombotic agents (16.3%, n=4,404) and lipid-modifying agents (55.2%, n=14,947; primarily atorvastatin and rosuvastatin [data not shown]).

### Anthropometric and clinical characteristics

*Table 3* shows the anthropometric and clinical characteristics of the study population.^19^ The mean body weight and height of enrolled subjects were 72.0 ± 13.0 kg and 161.5 ± 9.1 cm, respectively. The mean BMI was 27.0 ± 4.6 kg/m^2^, while the mean BF% was 32.0 ± 8.0%. Mean visceral fat percentage was 13.0 ± 6.5%. The mean waist and hip circumferences were 95.2 ± 10.5 cm and 100.5 ± 10.2 cm, respectively. Overall, 83.9% (n=31,828) of the study population had BMI above the normal range. About 15.4% (n=5,851) and 43.5% (n=16,508) were overweight and pre-obese as per BMI, respectively. One quarter (n=9,469) of the enrolled patients were obese, of which most (94.1%, n=8,917) were type 1 obese and 5.6% (n=534) were type 2 obese. Notably, despite a comparable mean BMI in both sexes, the mean BF% was higher among females (35 ± 7.8%) than males (29 ± 7.0%). Data on vital signs showed a mean systolic blood pressure of 131.3 ± 15.0 mmHg, diastolic blood pressure of 81.1 ± 8.4 mmHg and mean heart rate of 81.7 ± 9.80 beats per minute.

**Table 3:** Anthropometry and clinical characteristics of patients enrolled in the Indian Phenotype Registry^19^

Total population (N=37,927)			
Variables	Males	Females	Total
Anthropometric measure			
BMI, kg/m^2^ Range	27.0 (4.3) 12.2, 54.4	28.0 (4.9) 11.3, 55.0	27.0 (4.6) 11.3, 55.0
Body fat percentage, % Range	29.0 (7.0) 2.0, 68.0	35.0 (7.8) 2.0, 70.0	32.0 (8.0) 2.0, 70.0
Body weight, kg Range	74.0 (12.9) 45.0, 148.0	69.0 (12.4) 45.0, 134.7	72.0 (13.0) 45.0, 148.0
Height, cm Range	166.4 (7.3) 137.0, 200.0	155.4 (7.2) 135.0, 198.0	161.5 (9.1) 135.0, 200.0
Visceral fat percentage, % Range	14.0 (6.3) 2.0, 58.0	13.0 (6.8) 2.0, 64.4	13.0 (6.5) 2.0, 64.4
Waist circumference, cm Range	95.2 (10.2) 60.0, 130.0	95.2 (10.8) 60.0, 130.0	95.2 (10.5) 60.0, 130.0
Hip circumference, cm Range	99.1 (9.2) 80.0, 140.0	102.3 (11.0) 80.0, 140.0	100.5 (10.2) 80.0, 140.0
BMI category, n (%)^a^			
Underweight	467 (2.2)	577 (3.4)	1,044 (2.7)
Normal	3,150 (14.9)	1,905 (11.3)	5,055 (13.3)
Overweight	3,630 (17.2)	2,221 (13.2)	5,851 (15.4)
Pre-obese	9,558 (45.2)	6,949 (41.3)	16,508 (43.5)
Obese Obese type 1 Obese type 2 Obese type 3	4,293 (20.3) 4,094 (95.3) 192 (4.4) 7 (0.2)	5,175 (30.7) 4,822 (93.5) 342 (6.6) 11 (0.2)	9,469 (25.0) 8,917 (94.1) 534 (5.6) 18 (0.2)
Vital signs, mean (SD)			
SBP, mmHg Range	131.3 (14.8) 80.0, 200.0	131.2 (15.3) 80.0, 200.0	131.3 (15.0) 80.0, 200.0
DBP, mmHg Range	81.4 (8.4) 50.0, 142.0	80.8 (8.3) 50.0, 160.0	81.1 (8.4) 50.0, 160.0
Heart rate, beats/minute Range	81.4 (9.6) 50.0, 130.0	82.0 (9.9) 50.0, 130.0	81.7 (9.8) 50.0, 130.0
Diabetes-related measure			
HbA1c, % Range	8.3 (1.7) 5.5, 14.0	8.3 (1.7) 5.5, 14.0	8.3 (1.7) 5.5, 14.0
Duration of diabetes, n (%)			
<5 years	10,071 (47.7)	7,845 (46.6)	17,916 (47.2)
5–10 years	7,168 (33.9)	6,068 (36.1)	13,236 (34.9)
>10–20 years	3,152 (14.9)	2,518 (14.9)	5,670 (14.9)
>20 years	707 (3.3)	396 (2.3)	1,103 (2.9)
On-going anti-diabetic therapy, n (%)			
Yes	20,873 (98.9)	16,661 (99.0)	37,536 (99.0)
No	225 (1.1)	166 (1.0)	391 (1.0)

*Missing variables including data excluded due to erroneous or extreme values from the original sample: BMI (n=745), body fat percentage (n=746), body weight (n=696), height (n=1,515), visceral fat percentage (n=809), waist circumference (n=9,436), hip circumference (n=9,853), systolic blood pressure (n=1,617), diastolic blood pressure (n=1,619), heart rate (n=1,879), duration of diabetes (n=2) and HbA1c (n=2).*

*BMI categories (kg/m^2^): underweight = <18.5; normal = 18.5–22.9; overweight = 23.0–24.9; pre-obese = 25.0–29.9; and obese = ≥30.0.*

*Obese type 1 (obese), BMI = 30.0–40.0 kg/m^2^; obese type 2 (morbidly obese), BMI = 40.1–50.0 kg /m^2^; obese type 3 (super obese), BMI = >50.0 kg/m^2^.*

*^a^BMI category is based on ‘Asian criteria’.^19^*

*BMI = body mass index; DBP = diastolic blood pressure; HbA1c = glycated haemoglobin; SBP =systolic blood pressure; SD = standard deviation.*

**Table 4:** Body fat content by body mass index category^19^ A: Males

BMI category^a^	Body fat percentage					Essential fat (2–5%)	Athletes (6–13%)	Fitness (14–17%)	Acceptable (18–24%)	Obese (.25%)
	(N=40)	(N=405)	(N=571)	(N=4,010)	(N=16,285)
Underweight (n=202)	2 (1.0)	59 (29.2)	48 (23.8)	48 (23.8)	45 (22.3)
Normal (n=3,299)	13 (0.4)	122 (3.7)	262 (7.9)	1,760 (53.3)	1,142 (34.6)
Overweight (n=3,722)	6 (0.2)	79 (2.1)	81 (2.2)	1,087 (29.2)	2,469 (66.3)
Pre-obese (n=9,719)	14 (0.1)	130 (1.3)	140 (1.4)	941 (9.7)	8,494 (87.4)
Obese (n=4,369)	5 (0.1)	15 (0.3)	40 (0.9)	174 (4.0)	4,135 (94.6)

*Values are n (%).*

*BMI categories: underweight = <18.5 kg/m^2^; normal = 18.5–22.9 kg/m^2^; over weight = 23.0–24.9 kg/m^2^; pre-obese = 25.0–29.9 kg /m^2^; and obese = ≥30.0 kg/m^2^.*

*^a^BMI category is based on ‘Asian criteria’.^19^*

*BMI = body mass index.*

**Table 4:** B: Females

	Body fat percentage				
BMI category^a^	Essential fat (10–13%)	Athletes (14–20%)	Fitness (21–24%)	Acceptable (25–31%)	Obese (.32%)
	(N=123)	(N=642)	(N=939)	(N=2,834)	(N=12,085)
Underweight (n=110)	23 (20.9)	28 (25.5)	11 (10.0)	30 (27.3)	13 (11.8)
Normal (n=2,000)	36 (1.8)	284 (14.2)	286 (14.3)	524 (26.2)	837 (41.9)
Overweight (n=2,290)	19 (0.8)	118 (5.2)	219 (9.6)	494 (21.6)	1,425 (62.2)
Pre-obese (n=7,074)	34 (0.5)	159 (2.2)	349 (4.9)	1,410 (19.9)	5,085 (71.9)
Obese (n=5,249)	11 (0.2)	53 (1.0)	74 (1.4)	376 (7.2)	4,725 (90.0)

*Values are n (%).*

*BMI categories: underweight = <18.5 kg/m^2^; normal = 18.5–22.9 kg /m^2^; overweight = 23.0–24.9 kg/m^2^; pre-obese = 25.0–29.9 kg/m^2^; and obese = ≥30.0 kg /m^2^.*

*^a^BMI category is based on ‘Asian criteria’.^19^*

*BMI = body mass index.*

### Diabetes-related measures

The mean duration of diabetes was 78.2 ± 72.1 months, with more than half of patients (52.7%, n=20, 009) having diabetes for >5 years. Overall, the mean HbA1c was 8.3 ± 1.7%, with a similar distribution across both sexes. Most patients (99.0%, n=37,536) were taking an on-going anti-diabetic medication. Among all anti-diabetic medications, metformin monotherapy (97.9%, n=36,748) was the most commonly prescribed, followed by glimepiride (53.1%, n=19,944). Some patients were receiving newer anti-diabetic medications, such as sodium–glucose co-transporter-2 inhibitors (SGLT2is; dapagliflozin 16.2% [n=6,076], empagliflozin 4.2% [n=1,592]) and dipeptidyl peptidase-4 inhibitors (DPP4is; teneligliptin 25.5% [n=9,578] and sitagliptin 11.5% [n=4,298]). In addition, 15.8% (n=5,918) were receiving insulin and analogues (data not shown).

### Body fat content and body mass index categories

Among people with an underweight (2.7%, n=299) or normal BMI (13.3%, n=5,055), the mean BF% was 20.1% and 25.6%, respectively. Among people who were overweight (15.4%, n=5,851), pre-obese (43.5%, n=16,508) and obese (25.0%, n=9,469), the mean BF% was 28.8%, 32.1% and 37.3%, respectively.

### Sex-stratified subgroup analysis

The sex-stratified analysis revealed cases of NWO (BMI within the normal range and a high BF%). The subgroup analysis of males (n=21,098) revealed that, although 20.7% (n=4,369) had an obese BMI (most were type 1 obese), more than three- quarters (77.2%, n=16,285) had an obese BF%. Despite having a low BMI (underweight category, n=202), 22.3% (n=45) were obese as per their BF% (*Table 4A*).^19^ Similarly, more than one-third (34.6%, n=1,142) of males with normal BMI (n=3,299) and 66.3% (n=2,469) with an overweight BMI (n=3,722) had an obese BF%. Furthermore, nearly one quarter (23.8%; n=48) of males with an underweight BMI and more than half (53.3%, n=1,760) with a normal BMI had a relatively higher BF%, which was in the ‘acceptable’ category. Conversely, 12.6% (n=1,225) of males with a pre-obese BMI and 2.4% (n=234) with an obese BMI had a normal BF% (range 2–24%).

**Figure 1:**
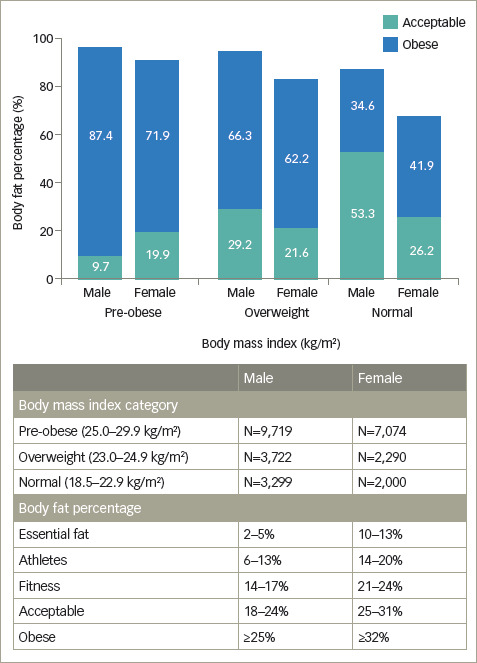
Characterization of Indian phenotype with lower body mass index and higher body fat percentage

*Note: Figure has other body fat categories; hence, percentages do not add up to 100%.*

Likewise, among females (n=16,827), 31.2% (n=5,249) had obesity per their BMI; however, a higher proportion (71.8%, n=12,085) were classified as obese per their BF%. Among females with an underweight (n=110) and normal (n=2,000) BMI, 11.8% (n=13) and 41.9% (n=837), respectively, had an obese BF%. Most (62.2%, n=1,425) females with an overweight BMI were classified obese. Moreover, 27.3% (n=30) with an underweight BMI and 26.2% (n=524) with a normal BMI had a BF% of 25–31%, resulting in them being grouped in the ‘acceptable’ category (*Table 4B*).^19^ *Figure 1* characterizes IP among individuals with a normal, overweight or pre-obese BMI, with a BF% in ‘acceptable’ or ‘obese’ category. Conversely, 27.6% of females (n=1,952) with a pre-obese BMI and 9.8% (n=514) with an obese BMI had a normal BF% (range 10–31%).

### Correlation between body fat percentage and body mass index

**Figure 2:**
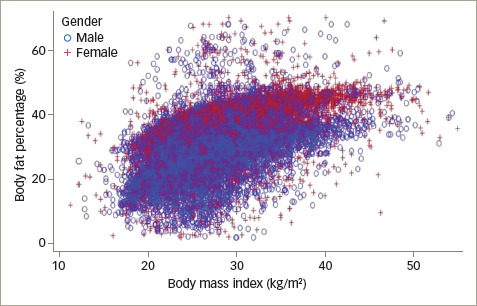
Correlation between body fat percentage and body mass index

A statistically significant, moderate positive correlation (r=0.51; p<0.0001) between BF% and BMI was seen in the overall population. Similar findings were reflected for both males and females, with a significant positive relationship between BF% and BMI in both groups (*Figure 2*). The scatter plot illustrates that even patients at the lower end of the spectrum of BMI tend to have a high BF%.

### Correlation between glycated haemoglobin level and body mass index categories

Among the patients with HbA1c <7.0%, nearly one quarter (24.9%, n=2,144) were obese, while 44.6% (n=3,835) were pre-obese (*Table 5*).^19^ Similar trends were observed for higher HbA1c levels; among patients with high HbA1c levels (≥7.0), the proportion of obese patients ranged from 25.0% to 26.1%, while that for pre-obese patients ranged from 43.0% to 45.4% (*Table 5*). However, the correlation analysis did not demonstrate any relationship between HbA1c level and BMI (*Supplementary Figure 1*).

*Pearson correlation coefficient (r) between body fat percentage and body mass index in the overall population; r=0.51, p<0.0001.*

*Males = 0.50, p<0.0001.*

*Females = 0.51, p<0.0001.*

### Body fat content with increasing age and diabetes duration

The sex-stratified subgroup analysis among males and females showed a significant association between BF% and age (*Supplementary Table 1*). Most males with an obese BF% were aged >60 years; most female subjects with an obese BF% were aged 50–59 years. Similarly, a strong significant association was found between BF% and duration of diabetes (p<0.05) in both sexes. Most males and females with an obese BF% had a duration of diabetes between 10 and 20 years (*Supplementary Table 2*).

## Discussion

This pan-India registry presents a comprehensive real-world reflection of the IP in patients with T2D. The study validates that Indians have a high BF%, despite having relatively lower BMI. Among the patients with normal BMI, many had an obese BF% despite using the Asia-specific BMI cut off, which is lower than that used for Caucasians.^22^ Of these patients with NWO, more females had an obese BF% than males. The correlation coefficient revealed a moderate positive relation between BMI and BF% in both males and females. Overall, the most prevalent comorbidities were hypertension and dyslipidaemia. Although most were taking on-going anti-diabetic medications, the mean HbA1c levels were higher than those recommended by the American Diabetes Association guidelines.^21^ There was a similar distribution of individuals with an obese and pre-obese BMI across HbA1c categories.

This study provides robust evidence confirming one of the crucial traits of the IP, which is NWO encompassing high BF% despite lower BMI among males and females. Among the patients with normal BMI, one-third of both sexes had an obese BF%, and among patients with a pre-obese BMI, more than two-thirds had an obese BF%. The relationship was further substantiated by a statistically significant positive correlation between BMI and BF%. Generally, in South Asians compared with Caucasians, BF% is 3–5 percentage points higher for the same BMI, and BMI is 3–4 units lower.^22,23^ South Asians tend to have earlier onset of diabetes, a longer duration of diabetes, lower BMI, lower waist circumference, lower HDL, but relatively higher triglycerides and HbA1c when compared with white Europeans.^24^ A study among young, healthy male adults in Indonesia revealed that insulin resistance is more strongly correlated with BF%, visceral fat and body weight than with BMI and waist circumference.^25^ Bhopal postulated a four-stage model explaining the higher risk of T2D in South Asian compared with European populations.^26^ In South Asians compared with Europeans: (1) at birth, babies are smaller, have lower adipose and lower lean mass; (2) in childhood and early adulthood, excess calorie intake deposits preferentially in the upper body and ectopic fat stores (rather than lower body or as superficial subcutaneous fat); (3) a vicious cycle of high levels of plasma insulin, triglycerides and glucose, and a fatty liver appears, exacerbated by low physical activity and excess calories; (4) pancreatic β cells fail due to fewer β cells at birth, exposure to apoptotic triggers such as fat in the pancreas, and high demand from insulin resistance.^26^

**Table 5:** Glycated haemoglobin distribution by body mass index category^19^

	HbA1c category			
BMI category^a^	<7.0% (N=8,602)	7.0–<8.0% (N=10,175)	8.0–<9.0% (N=7,721)	≥9.0% (N=10,684)
Underweight	61 (0.7)	89 (0.9)	56 (0.7)	93 (0.9)
Normal	1,217 (14.1)	1,360 (13.4)	943 (12.2)	1,535 (14.4)
Overweight	1,345 (15.6)	1,604 (15.8)	1,207 (15.6)	1,695 (15.9)
Pre-obese	3,835 (44.6)	4,581 (45.0)	3,502 (45.4)	4,590 (43.0)
Obese	2,144 (24.9)	2,541 (25.0)	2,013 (26.1)	2,771 (25.9)
Obese type 1 (obese)	2,019 (94.2)	2,388 (94.0)	1,905 (94.6)	2,605 (94.0)
Obese type 2 (morbidly obese)	120 (5.6)	146 (5.7)	105 (5.2)	162 (5.8)
Obese type 3 (super obese)	5 (0.2)	7 (0.3)	3 (0.1)	4 (0.1)

*Values are n (%).*

*BMI categories: underweight = <18.5 kg/m^2^; normal = 18.5–22.9 kg/m^2^; overweight = 23.0–24.9 kg/m^2^; pre-obese = 25.0–29.9 kg/m^2^; and obese = ≥30.0 kg/m^2^.*

*^a^BMI category is based on ‘Asian criteria’.^19^*

*BMI = body mass index; HbA1c = glycated haemoglobin.*

A study from Sri Lanka demonstrated a significant positive correlation between BMI and BF% in males (r=0.75, p<0.01) and females (r=0.82, p<0.01) of all ages.^27^ The paradox of low BMI and high BF% was starkly reported for Indians in Singapore, with Indians having the highest BF% among a mixed population of Indian, Chinese and Malayan people.^28^ NWO is an under-recognized arena; however, evidence on its pathophysiology and its association with metabolic diseases such as T2D, hypertension and dyslipidaemia is evolving.^29^ Results from the Kerala Diabetes Prevention Program demonstrated that about one-third of the study subjects had NWO.^30^ The study also reported a significantly higher proportion of individuals with T2D, hypertension and dyslipidaemia in the NWO group compared with the non-obese group.^30^ NWO was also identified as an independent strong predictor of cardiovascular mortality, and a widely prevalent problem in individuals of Asian descent.^29^ A study among males in Lucknow reported that 44.0% of subjects showed a high BF% (>25%) with a BMI of 24.0–24.9 kg/m^2^, and 4.7% at a lower BMI (<20 kg/m^2^). Rates of high BF% in the BMI range 20–21.9 kg/m^2^ and 22–23.9 kg/m^2^ were 9.5% and 18.4%, respectively. In addition, BMI was highly correlated with BF% (r=0.73, p<0.001).^17^

The study results demonstrate that females have a proportionally higher BF% than males, despite having similar BMI. A real-world study including data from the Korea National Health and Nutrition Examination Survey (2007–2010) revealed that whole BF% content was higher in women than in men.^31^ This study also demonstrated that the average BMI and whole BF% content of women were higher than those of men; however; the average waist circumference at the time of diabetes diagnosis was similar (approximately 88 cm) in both sexes.^31^ Similarly to our results, a community-based study involving 1,080 adult participants from Haryana, India, reported a comparable mean BMI between males and females, but with a higher mean BF% in females (28.69%) than in males (26.02%). The study further showed a strong positive correlation between BMI and BF% (r=0.747, p<0.001) in the overall population.^32^

A cross-sectional study among non-pregnant women from Haryana revealed that women with underweight and normal BMI, had mean (SD) BF% of 23.8% (4.1) and 31.0% (5.0), respectively. Also, there was a strong positive relation between BMI and BF% (r=0.85, p<0.001).^32^ A study among adolescents (aged 10–14 years) in Dibrugarh, India, showed that of the participants with normal BMI, 9% were overweight and 1% were obese under the BF% criteria. In addition, BMI and BF% had a significant positive correlation (r=0.70, p<0.001).^33^ According to the thrifty genotype hypothesis, the predisposition to diabetes must have evolved as an adaptive trait in certain environmental situations, which later turned disadvantageous because of the changes in lifestyle.^34^ Early prevention or treatment of childhood obesity focusing on lifestyle factors may be critical for preventing diabetes in South Asians. Chooi et al. evaluated the effects of diet-induced 5% weight loss on body composition in metabolically obese normal-weight Asians, and revealed that weight loss decreases total fat mass by ~9% and intrahepatic fat by ~50% (p<0.05). Fasting plasma insulin and cardiometabolic factors, such as triglyceride and LDL, HDL and total cholesterol concentrations, were also reduced (p<0.05). Additionally, insulin sensitivity indices increased by 21% to 26% (both p< 0.05).^35^

More than half of this study's patients were aged 40–60 years. Diabetes occurs at a younger age and a lower BMI in South Asians compared to Caucasians, raising the risk of cardiovascular and renal complications.^36^ A registry including data from Singapore (including Indians) elucidated that diabetes was three times more common in Southeast Asians compared with white patients with heart failure, despite younger age and less obesity.^37^ Interestingly, ethnic differences in T2D risk between South Asians originate in childhood. A study of 4,633 children (9- to 10-year-olds) of South Asian, black African-Caribbean and white European origin, reported that South Asian children showed stronger associations with adiposity, insulin resistance and HbA1c than white Europeans. Fat mass was positively associated with HbA1c in South Asians and black African-Caribbeans, but not in white Europeans; for a 1×SD increase in fat mass percentage, percentage differences in HbA1c were 0.04% (95% confidence interval [CI]: 0.03–0.06), 0.04% (95% CI 0.02–0.05) and 0.02% (95% CI -0.00–0.04), respectively (p interaction <0.001).^38^

BF% is a crucial element in predicting T2D, with marked differences between sexes. A study from India revealed that despite an insignificant correlation between HbA1c levels and BMI, there was a significant positive correlation between HbA1c and fat mass (r=0.452, p<0.001) in patients with T2D.^16^ In addition, a prospective study from India also reported that centrally and peripherally obese subjects with dyslipidaemia had a significant association with HbA1c in T2D.^39^ A community-based Korean cohort study demonstrated that, compared with people with a lower BF% (quintile 1), the risk for T2D significantly increased among those with a higher BF% (22.8% in men and 32.9% in women; ≥ quintile 4).^40^ However, our study showed a similar distribution for obese and pre-obese individuals with respect to BMI across the different HbA1c levels.

There is a significant loss of skeletal muscle mass and an increase in BF% with increasing age; the term ‘sarcopenia’ relates to age-related decreases in muscle mass and strength. Low muscle mass and increased BF% are associated with a risk of developing metabolic disorders, including T2D.^41^ Because of lifestyle changes and longer life expectancy, the burden of T2D and sarcopenic obesity is projected to increase globally; both share common risk factors, such as ageing and general obesity.^42^ Individuals with T2D tend to develop sarcopenic obesity, which is likely to increase with age.^43^ A Japanese study reported that patients with diabetes had higher risk of sarcopenia than patients without diabetes. Additionally, elderly sarcopenic males had significantly lower BF% and a longer duration of T2D compared with non-sarcopenic males (p<0.01).^44^ Another recent study reported increased odds of sarcopenia with increased percentage of total fat in individuals with T2D compared with the control group (men: odds ratio [OR] 1.31, 95% CI 1.10–1.75; women: OR 1.18, 95% CI 1.03–1.43).^45^ Our study did not evaluate muscle mass and strength; however, we did find a strong association between an obese BF% and increasing age, and duration of diabetes.

Metformin monotherapy was the most commonly prescribed (97.9%) oral anti-diabetic drug in this study; however, some patients were receiving newer oral anti-diabetic drugs such as SGLT2is and DPP4is. The Research Society for the Study of Diabetes in India–Endocrine Society of India 2020 clinical practice recommendations for management of T2D in India suggest that lifestyle changes (including dietary modification, exercise and behavioural management) alongside pharmacotherapy and bariatric surgery are the most effective interventions for weight management in patients with T2D.^46^ The guidelines recommend novel therapeutic agents such as glucagon-like peptide (GLP)-1 agonists, DPP4is and SGLT2is as add-ons to metformin in obese patients with T2D. The pleiotropic effect of SGLT2is and GLP-1 agonists can facilitate weight management, particularly by reducing visceral fat.^47,48^ SGLT2is and GLP-1 agonists minimize weight gain when added to metformin and/or sulfonylurea, and the clinically meaningful body weight reductions can further contribute reduced HbA1c and systolic blood pressure.^49,50^ The co-administration of these novel oral anti-diabetic drugs that target complementary mechanisms represents an effective strategy for weight loss, with additional cardiorenal benefits among South Asian people.^51^

The Indian Phenotype Registry is a real-world registry based on data collected from routine clinical practice, with no follow-up visits. Hence, issues related to an observational registry, such as loss to follow-up and missing data, as well as the unavailability of zone-specific data, form some important limitations. Data on dyslipidaemia, such as HDL, LDL, total cholesterol and triglycerides, were not collected in this study. Although we assessed the correlation between HbA1c and BMI, the correlation between HbA1c and BF% was not investigated. Additionally, being a cross-sectional analysis, the study cannot affirm a causal association between obesity and other variables. However, this is one of the largest registries worldwide exploring IP characteristics. In India, the relationship between BMI and BF% has been investigated in region-specific prevalence studies, but with smaller sample sizes. Results from the IP registry can augment and substantiate the current evidence pool describing the Asian phenotype. The large sample size, with a representative population from diverse geographies and healthcare tiers of India, strengthen the results of the study.

## Conclusion

The Indian Phenotype Registry is a pan-India cross-sectional registry that aims to generate nationwide data and provide clear insights about the phenotypic characteristics specific to Indian patients with T2D. Results from this study affirm the key characteristics of the IP of a low BMI with a high BF%. Additionally, the mean HbA1c levels were high, despite the majority of patients receiving anti-diabetic medications. Insights on the high BF distribution in Indian patients with T2D highlight the importance of effectively identifying risk factors (primordial prevention), diagnosing early (primary prevention) and aggressively managing obesity with intensive diet, exercise and therapy interventions to reduce complications and comorbidities (secondary prevention). These findings will guide therapeutic decisions on the choice of agents for glycaemic control, with preference for drugs that promote weight loss, such as SGLT2is and GLP-1 agonists, or are weight neutral, such as metformin, α-glucosidase inhibitors and DPP4is.^47–50^
